# Comparison of GeneXpert and line probe assay for detection of *Mycobacterium tuberculosis* and rifampicin-mono resistance at the National Tuberculosis Reference Laboratory, Kenya

**DOI:** 10.1186/s12879-019-4470-9

**Published:** 2019-10-15

**Authors:** S. A. Aricha, L. Kingwara, N. W. Mwirigi, L. Chaba, T. Kiptai, J. Wahogo, J. S. Otwabe, P. O. Onyango, M. Karanja, C. Ayieko, S. W. Matu

**Affiliations:** 1 School of Physical and Biological Sciences, Maseno University, Kisumu, Kenya; 2 National Public Health Laboratories, Nairobi, Kenya; 3 Amref Health Africa, Nairobi, Kenya; 4 Kenya Medical Research Institute, Nairobi, Kenya; 5 Strathmore University, Nairobi, Kenya; 6 National AIDS and STI Control Program, Nairobi, Kenya; 7 Kisii teaching and Referral Hospital, Nairobi, Kenya

**Keywords:** LPA, GeneXpert, Sensitivity, Specificity, Drug-resistant TB

## Abstract

**Background:**

The dual challenge of low diagnostic sensitivity of microscopy test and technical challenge of performing a TB culture test poses a problem for case detection and initiation of Tuberculosis (TB) second-line treatment. There is thus need for a rapid, reliable and easily accessible assay. This comparative analysis was performed to assess diagnostic performance characteristics of GeneXpert MTB/RIF and Line Probe Assay (LPA).

**Methods:**

Three hundred twenty nine sputum samples of patients across the 47 counties in Kenya suspected to have drug resistant TB were picked and subjected to GeneXpert, LPA and Culture MGIT at the National TB Reference Laboratory. Sensitivity, specificity and predictive values were then determined to assess the performance characteristics of the various assays.

**Results:**

Against culture MGIT as the gold standard for TB diagnosis, GeneXpert had a sensitivity, specificity, positive predictive value, and negative predictive value of 78.5, 64.9, 59.4 and 82.2% respectively while LPA had 98.4, 66.0, 65.4 and 98.4%. For diagnosis of rifampicin mono-resistance GeneXpert had a moderate agreement (Kappa 0.59, *P* < 0.01) (sensitivity 62.50%, specificity 96.50%) while LPA that had almost perfect agreement (Kappa = 0.89, *p* < 0.01) with a (sensitivity 90.0% and specificity 99.1%).

**Conclusion:**

LPA has a better performance characteristic to GeneXpert and an alternative to culture with regards to detection of RIF’s mono-resistance.

## Background

Tuberculosis (TB) causes substantial morbidity and mortality [1]. About 10.0 million TB cases and 1.4 million deaths worldwide were reported in 2018 [2]. In the same year, 480,000 cases of multidrug-resistant TB (MDR-TB) and 100,000 cases of rifampicin monoresistant TB were reported. Kenya is among the 22 countries with the highest TB burden globally, with a national TB case prevalence of 558 per 100,000 people as per the Kenya National TB prevalence survey 2016 [3, 4]. A key component in TB control and management is rapid diagnosis [5]. Tuberculosis diagnosis is routinely done using microscopy in middle and low-income countries since it is readily available. Microscopy identifies *Mycobacterium tuberculosis* bacilli which is treatable with first-line Anti-TB drugs. Its sensitivity in TB detection is at 60% as per the Kenya national TB case prevalence survey of 2016 resulting into a high number of missed cases. This results into a continuous transmission especially among the missed cases. Moreover TB microscopy has a low sensitivity in the detection of TB in children and patients with extra- pulmonary TB [1].

Drug resistance TB is transmissible and results mainly from sub-optimal TB treatment. The diagnostic tools available for its detection are limited and often less sensitive and poorly adaptable for poor resource settings [1]. This results to large proportion of drug resistance TB cases remaining undiagnosed leading to continuous transmission and increased cost of treatment and management. Currently, TB culture remains the gold standard for drug resistance TB diagnosis although it is more expensive and takes not less than 4 weeks to get results [6].

The molecular-based methods detect *Mycobacterium tuberculosis* nucleic acid materials in sputum samples and the mutations resulting into anti TB drugs resistance. GeneXpert and LPA methods are both capable of detecting mutations causing resistance against Rifampicin. In addition, LPA can also detects mutations related to isoniazid drug [5]. Though widely distributed in the Kenyan health care system, the performance of these two assays have not been evaluated to determine their performance in the detection of TB and associated drug resistance.

Early diagnosis of TB resistance is important for optimal management of drug-resistant cases; this necessitates rapid drug susceptibility testing in the management of MDR/XDR TB [6, 7]. The standard DST test is a twostep procedure involving culture and sensitivity testing. This takes a long time, first to isolate a culture and then to perform drug susceptibility testing (indirect DST). If DST could be set up at the same time as when a processed specimen is inoculated in solid and or liquid medium (direct DST), it could save significant time for the detection of drug resistance.

In Kenya, direct DST using the conventional solid medium has been established [8, 9]. The only disadvantage is that it takes a long time to obtain results on solid medium, as the growth rate on such media is lower. This impacts negatively to critical patients who are in need of a shorter result turnaround time for patient management.

The study thus determined the sensitivity, specificity, positive and negative predictive values of GeneXpert and LPA in TB diagnosis using liquid culture as the gold standard. In addition, we assessed the sensitivity, specificity, positive and negative predictive values of GeneXpert and LPA in Rifampicin mono resistance detection using liquid culture as the gold standard.

## Methods

We analysed 306 samples between November 2016 and March 2017. A systematic random sampling procedure was used to select sputum samples from a pool received from various diagnostic sites located in the 47 counties. An average of ten samples was picked from all samples received at NTRL during each day for a period of 33 days to obtain 329 samples. To ensure equal chance of every sample received being selected, all the samples received in a day were divided by 10 to get the interval of picking.

### Sample collection and transportation

Suspected MDR-TB patients from clinical diagnosis and patient result history review were given a sputum collection container with instructions on how to collect and deliver a sputum sample to the facility. The sputum sample together with the patient request form from each diagnostic site was packed in a standard triple packaging container and an ice bag inserted to keep the sample at 2-8 °C. All the samples were transported by a registered courier to NTRL for culture, drug susceptibility and molecular diagnostic testing.

### Sample reception and processing

At NTRL, sputum samples together with the lab request form were received and checked for completeness in filling the laboratory request form, correct sample tube labelling and leakage. Those accepted were given laboratory number for processing. All acceptable sputum samples were decontaminated using the *N*-acetyl-l-cysteine-sodium citrate-NaOH (NALC-NaOH) method. Samples were decanted following centrifugation at 3000 g for 15 min, and the pellets were re-suspended to make 3 ml using phosphate buffer solution. Four 0.5 ml aliquots from each sample were used in florescent microscopy, MGIT960 culture, GeneXpert, and LPA. Another 1 ml aliquot of each sample was stored at − 80 °C as back up.

### MGIT960 culture and drug susceptibility testing (SIRE MGIT-DST)

A 500-μl aliquoted sample were incubated in *Mycobacterium* growth indicator tubes (MGIT) in the BACTEC machine (BACTEC™ MGIT™ 960 System, Series 1300 A2, Becton Dickson and Company, MA, USA) for a maximum of 42 days (6 weeks) from initial incubation date. The MGIT tubes in the BACTEC machine were flagged by green light for no growth and red light for growth of *Mycobacterium*, on the front drawer of the BACTEC MGIT 960 machine. Cultures that showed growth were confirmed for *Mycobacterium tuberculosis* by use of brain heart infusion agar (BHI), Ziehl Neelsen stain and capillia technique and subjected to drug susceptibility testing (DST) against Isoniazid, Rifampicin, Streptomycin and Ethambutol (SIRE). To control tubes, 0.5 ml of Growth Control working solution was added into labelled drug free Mycobacterium growth indicator tubes, and to all the other drugs containing tubes labelled S (streptomycin), I (isoniazid), R (rifampicin) and E (ethambutol). When the growth control tubes reached a growth unit of 400 or more the instrument indicated that the test was complete and interpreted the results as resistant or susceptible. The tubes were removed from the machine after being scanned and printed. The final pattern of susceptibility testing was manually interpreted per sample as fully susceptible, mono-resistant, poly-resistant or multidrug resistant. To assure the test quality a set of H37RV controls were included in all the run.

### Line probe assay

The line probe assay (LPA), based on strip technology was used to diagnose TB and detect RIF as well as Isoniazid (INH) resistance due to mutations in *rpoβ*, and both *inhA* and *katG* genes. The test was performed according to the manufacturer’s protocol (Hain Life Science GmbH, Nehren, Germany). The method involved three processes: DNA extraction, multiplex PCR amplification, and reverse hybridization. The DNA strip was removed from the tube and marked as per the number of samples. It was then added to each well containing 20 μl of corresponding amplified DNA sample with coloured part facing up. The well was placed in the twincubator and hybridization procedure was initiated. Hybridization occurred by pre-warming the hybridization buffer to 45 °C in water bath for 15 min in the twincubator machine (Hain Life Science GmbH, Nehren, Germany). Denaturing solution, 20 μl was pipetted to each of the tray that was used, and then 1 ml of rinse solution added per well and incubated for 1 min. The well was removed and rinsed with a rinse solution. One millilitre of the conjugate was added into each well, then incubated for 30 min, removed and washed with rinse solution. Finally, 1 ml of the substrate was added into the well and incubated for 10 min and then washed twice with distilled water. The strips were then left to dry and results scanned and interpreted by the Hain Life Science GmbH, Nehren, Germany machine as either *Mycobacterium tuberculosis* detected, resistant, sensitive or invalid.

### GeneXpert MTB/RIF

The GeneXpert MTB/RIF (Cepheid, Sunnyvale, CA) test was performed as per the manufacturer’s instruction. Aliquots of decontaminated samples were taken out of 4 °C storage, together with the sample reagent buffer containing NaOH and isopropanol were mixed at the ratio of 1:3 followed by incubation at room temperature for 15 min. Two milliliters of the sample were then transferred into the GeneXpert MTB/RIF cartridge (Cepheid, Sunnyvale, CA) and loaded into the GeneXpert MTB/RIF (Cepheid, Sunnyvale, CA). The results were generated after 2 hours, reported as both *M. tuberculosis* negative or positive, and whether those positive were RIF susceptible or resistant.

### Ethical consideration

Ethical approval for this study was obtained from Amref Health Africa Ethics and Scientific Review committee. The samples were assigned unique study codes and delinked with the patient maintaining age and sex as the only socio-demographic data. The study did not alter the original patient results in any way.

### Statistical analysis

Categorical and numerical variables were summarized using frequency (percentage) and mean (SD) respectively. Sensitivity, specificity and predictive values were calculated to compare the performances between tests. Agreements of different tests were assessed by Cohen’s Kappa statistics. A test with *P* ≤ 0.05 was considered statistically significant. The precision of the estimates was reported using 95% confidence intervals (95% CI). Statistical analyses were done using R.

## Results

Three hundred and twenty-nine sputum samples of patients suspected to have drug resistant TB were picked and subjected to GeneXpert, LPA and Culture MGIT. Out of 329 samples processed, 23 samples were excluded; 10 had *Mycobacterium* other than *M. tuberculosis*; 4 were contaminated; 4 were invalid; 3 had errors; 2 were indeterminate. The remaining three hundred and six (306) samples were analysed. Of the 306 samples, 108 (35.29%) were from female and 198 (64.71%) from male participants. The mean age of study participants who provided sputum samples used in this study was 36.86 (SD =13.3) years. Of the 306 samples tested for smear concentration microscopy, 39.2% were smear negative (). For the smear positive samples, the following bacillary loads were observed: 1+ in 48 (15.7%), 2+ in 56 (18.3%) and 3+, 41 (13.4%). 4B, 5B and 10B were observed in just 5 samples while 6B results were observed in 36 (11.8%) samples (Table 1).

**Table 1:** Distribution of participants’ Age, Gender, and Smear concentration microscopy results

	FREQUENCY/MEAN	PERCENT/SD
AGE	Mean is 36.8	13.3
GENDER		
FEMALE	108	35.3
MALE	198	64.7
SMEAR CONCENTRATION		
1+	48	15.7
2+	56	18.3
3+	41	13.4
4B	1	0.3
5B	3	1.0
10B	1	0.3
NEG	120	39.2
6B	36	11.8

### Diagnosis of tuberculosis

To compare the performance of GeneXpert and LPA on smear positive results, 186 samples were used. GeneXpert detected 142 (76.5%) and LPA detected 167 (89.8%) samples as positive. In terms of sensitivity, specificity, and predictive values, GeneXpert recorded sensitivity of 62.2% and specificity of 43.2% with positive and negative predictive value of 79.0 and 28.4% respectively. LPA reported sensitivity and specificity of 99.2 and 26.9% respectively with positive and negative predictive value of 70.7 and 94.7% respectively (Tables 2 and 3). Of 306 samples tested, Culture MGIT detected *M.tuberculosis* in 121 (39.5%) samples, GeneXpert detected *M.tuberculosis* in 160 (52.3%), and LPA detected *M.tuberculosis* in 182 (59.5%). (Table 4). Using Culture MGIT as the gold standard, the GeneXpert correctly identified *M. tuberculosis* in 95 of 121 Culture MGIT positive samples while LPA correctly identified *M. tuberculosis* in 119 of 121 Culture MGIT positive samples. These translate to sensitivity, specificity, positive predictive value, and negative predictive value of 78.5, 64.9, 59.4 and 82.2% respectively for the GeneXpert and 98.4, 66.0, 65.4 and 98.4% respectively for the LPA **(**Table 5**)**.

**Table 2:** M. tuberculosis detection by GeneXpert and LPA against Culture MGIT using smear positive samples

	Smear positive		
	Detected (*n* = 119)	Not Detected (*n* = 67)	Total (=186)
Gene Xpert			
Detected	94	48	142 (76.3%)
Not Detected	25	19	44 (23.7%)
LPA			
Detected	118	49	167 (89.8%)
Not Detected	1	18	19 (10.2%)

**Table 3:** Performance of GeneXpert and LPA tests compared to Culture MGIT in detecting M. tuberculosis using smear positive samples

	Sensitivity (%)	Specificity (%)	Predictive values (%)		Kappa
			Positive	Negative	
*Gene Expert*	66.2 (57.8–73.9)	43.2 (28.4–59.0)	79 (73.9–83.3)	28.4 (20.8–37.4)	0.079 (*P* = 0.257)
*LPA*	99.2 (95.4–100.0)	26.9 (16.8–39.1)	70.7 (67.5–73.6)	94.7 (71.1–99.3)	0.309 (*p* < 0.001)

**Table 4:** M. tuberculosis detection by GeneXpert and LPA against Culture MGIT

	Culture MGIT		
	Detected (*n* = 121)	Not Detected (*n* = 185)	Total (=306)
Gene Xpert			
Detected	95	65	160 (52.3%)
Not Detected	26	120	146 (47.7%)
LPA			
Detected	119	63	182 (59.5%)
Not Detected	2	122	124 (40.5%)

**Table 5:** Performance of GeneXpert and LPA tests compared to Culture MGIT in detecting M. tuberculosis

	Sensitivity (%)	Specificity (%)	Predictive values (%)		Kappa
			Positive	Negative	
GeneXpert	78.5 (70.1–85.5)	64.86 (57.5–71.7	59.4 (54.1–64.5)	82.2 (76.4–86.8)	0.411 (*P* < 0.01)
LPA	98.4 (94.2–99.8)	66.0 (58.6–72.7)	65.4 (60.7–69.8)	98.4 (93.9–99.6)	0.591 (*p* < 0.01)

The 121 Culture MGIT positive samples were subjected to the drug susceptibility testing (DST) against Isoniazid, Rifampicin, Streptomycin and Ethambutol. 9 (7.4%), 12 (9.9%), 10 (8.3%), and 7 (5.8%) samples were found to be resistant to Streptomycin, Isoniazid, Rifampicin and Ethambutol respectively. 4.1% of the samples were resistant to all the four antibiotics and 86.6 of the samples were sensitive to all the antibiotics. **(**Table 6**)**.

**Table 6:** Antibiotic resistant detection by DST MGIT on positive samples

*N* = 121		
	Frequency	Percent
DST_1ST_STREPTOMYCIN		
RESISTANT	9	7.4
SENSITIVE	112	92.6
DST_1ST_ISONIAZID		
RESISTANT	12	9.9
SENSITIVE	109	90.1
DST_1ST_RIFAMPICIN		
RESISTANT	10	8.3
SENSITIVE	111	91.7
DST_1ST_ ETHAMBUTOL		
RESISTANT	7	5.8
SENSITIVE	114	94.2
RESISTANT TO ALL GENES	5	4.1
SENSITIVE TO ALL GENES	105	86.8

For assessing the performance of GeneXpert and LPA tests in detecting Rifampicin and Isoniazid resistance, pairwise undetected results from the Culture MGIT, GeneXpert and LPA tests were excluded. Culture MGIT and GeneXpert were then compared in 95 samples while LPA and Culture MGIT were compared in 119 samples. Culture MGIT drug susceptibility testing was used as the gold standard. GeneXpert and LPA had a sensitivity value of 91.6% (Table 7). There was a moderate agreement (Kappa = 0.59, *P* < 0.01) between culture and Gene expert in detecting Rifampicin mono-resistance (sensitivity = 62.50%, specificity = 96.50%) and an almost perfect agreement (Kappa = 0.89, *p* < 0.01) between Culture and LPA (sensitivity = 90.0% and specificity = 99.1%) **(**Table 8). The agreement between Culture MGIT and LPA in detecting Isoniazid (KatG and INHA) resistance was substantial (Kappa = 0.758 *p* < 0.001) with sensitivity and specificity values being 91.7 and 95.3% respectively (Tables 9 and 10).

**Table 7:** Detection of rifampicin resistance by GeneXpert and LPA against Culture MGIT DST

	DST_1ST_RIF		
	*Resistant (n = 8)*	*Sensitive(n = 89)*	*Total (n = 95)*
GENEXPERT RIF			
RESISTANT	5	3	8 (8.4%)
SENSITIVE	3	84	87 (91.6%)
LPA RIF	*Resistant (n = 10)*	*Sensitive(n = 109)*	*Total (n = 119)*
RESISTANT	9	1	10 (8.4%)
SENSITIVE	1	108	109 (91.6%)

**Table 8:** Performance of Gene expert and LPA test as compared to DST in detecting RIF resistance

	Sensitivity	Specificity	Predictive values		Kappa
			Positive	Negative	
GeneXpert	62.5 (24.5–91.5)	96.6 (90.3–99.3)	62.5 (32.7–85.1)	96.6 (92.0 98.6)	0.59 (*P* < 0.01)
LPA	90.0 (55.5–99.8)	99.1 (95.0–100)	90.0 (55.9–98.5)	99.1 (94.4–99.9)	0.89 (*P* < 0.01)

**Table 9:** Detection of Isoniazid (INH) resistance by LPA against Culture MGIT DST

	DST_1ST_ISONIAZID		
	*Resistant (n = 12)*	*Sensitive(n = 107)*	*Total (n = 119)*
*KATG*			
RESISTANT	8	3	11 (9.2%)
SENSITIVE	4	104	108 (90.8%)
*NHA*			
RESISTANT	3	2	5 (4.2%)
SENSITIVE	9	105	114 (95.8%)
*KATG AND INHA*			
RESISTANT	11	5	16 (13.4%)
SENSITIVE	1	102	103 (86.6%)

**Table 10:** Performance of LPA test as compared to DST in detecting Isoniazid (INH) resistance

	Sensitivity	Specificity	Predictive values		Kappa
			Positive	Negative	
KatG	66.7 (34.9–90.1)	97.2 (92.0–99.4)	72.7 (44.9–89.7)	96.3 (92.1–98.3%)	0.663 (*P* < 0.01)
inhA	25.0 (5.5–57.19)	98.1 (93.4–99.8)	60 (21.7–89.01)	92.11 (89.4–94.2)	0.319 (*P* < 0.01)
KatG and inhA	91.7 (61.5–99.8)	95.33 (89.4–98.5)	68.8 (47.9–84.4)	99.0 (94.0–99.9)	0.758 (*p* < 0.01)

## Discussion

Resurgence and rapid spread of TB and drug resistant *M. tuberculosis* over the recent years raises an urgent need to find an efficient rapid assay for diagnosis and detection of drug resistant TB [10]. No study has been carried out in Kenya to compare the performance of both GeneXpert and LPA. In this study, the performance of GeneXpert MTB/RIF and LPA in detecting TB and Rifampicin mono-resistance was compared to that of liquid culture as the gold standard.

Using smear as the gold standard for diagnosing TB, GeneXpert MTB/RIF, had a diagnostic sensitivity, specificity, positive predictive value and negative predictive values comparable to the Thibela TB’ study that yielded a sensitivity, specificity, PPV and NPV of 59.8%, 49/7, 79.2 and 31.5% [11–13]. Similar studies have yielded similar results. LPA had a better diagnostic performance characteristic compared to GeneXpert when using smear microscopy as the gold standard with results comparable to the South African meta-analysis study which yielded a sensitivity, specificity, positive predictive value and negative predictive values of 99.1, 28.4, 69.9 and 94.6% respectively [14–18]. Studies in similar settings have also reported same results [19–21].

GeneXpert performance when MGIT was used as the gold standard, gave result similar to other studies that had Sensitivity, specificity, positive predictive value, and negative predictive values of MTB at 62.6%(55.2, 69.5), 99.6% (99.4, 99.7), 81.3% (73.9, 87.3), and 98.9 (98.6, 98.8), while Kappa agreement was 98.5% (98.2, 98.8). Similar results have also been seen among HIV-infected adults enrolling in an ART clinic whose sputum was tested by GeneXpert regardless of symptoms [21]. Our LPA findings compares to the sensitivity and specificity of LPA diagnostic platform in South African and South American was 81 and 100% respectively [22]. In India two studies showed a sensitivity and specificity of 96 and 99% respectively in 248 smear positive patients [13] and, sensitivity and specificity of 68.4 and 89.3% respectively from 213 sputum smear negative samples [23]. Similarly, a study in high risk MDR-TB population in Taiwan also reported a sensitivity and specificity of 85.9 and 65.7% respectively [24]. Also, in Ethiopia, a study on 274 presumptive MDR-TB patients both smear positive and negative, reported sensitivity and specificity of 96.4 and 100% from smear positive samples, 77.8 and 97.2% smear negative samples respectively [11]. It appears that failure to separate the samples as per the smear results did not affect the sensitivity but affected the specificity in this study.

There was a moderate agreement (Kappa = 0.59, *P* < 0.01) between culture and Gene expert in detecting Rifampicin resistance (sensitivity = 62.50%, specificity = 96.50%) when MGIT was used as the reference assay. This was comparatively lower than LPA that had almost perfect agreement (Kappa = 0.89, *p* < 0.01) between MGIT Culture and LPA (sensitivity = 90.0% and specificity = 99.1%) for detecting Rifampicin resistance and a substantial agreement (Kappa = 0.758 *p* < 0.001) with sensitivity and specificity values being 91.7 and 95.3% respectively for detecting in detecting Isoniazid (KatG and INHA) resistance.

GeneXpert MTB/RIF sensitivity, specificity, positive and negative predictive values for Rifampicin mono resistance detection of 62.50 and 96.50%, 62.50 and 96.60% respectively compares with a similar study in South Africa which recorded sensitivity and specificity of 79 and 94% respectively [25]. It was notable that our study reported lower sensitivity compared to the South African study and a similar one within the Asian population which had a pooled sensitivity of 68–100% [26]. Previous studies have suggested reasons for these, for example discrepancy in the volume of the sample discharged into the cartridge [27] and failure to separate samples based on smear results because smear positive samples have been reported with higher sensitivity when compared to samples which are smear negative [11].

The sensitivity of Rifampicin mono resistance using LPA observed in this study is corresponding to other similar studies. Two case examples from South African and South American population using the smear positive samples, had a sensitivity and specificity of 92 and 97% [22]. This is corresponding to similar findings from a study based on high risk MDR- TB set up of Taiwan which reported a sensitivity of 96% [24]. In addition, a study in New Delhi recorded a sensitivity and specificity of 97.6 and 94.4% when they used LPA in smear positive samples [28] similarly to the South Africa study reported a sensitivity and specificity of 97.7 and 91.8% respectively [29]. In other studies done in East African countries, the sensitivity and specificity of Rifampicin mono resistance detection was 96.4 and 100% among smear positive samples and 77.8 and 98.2% among smear negative samples in Ethiopia [11] and 100 and 96.1% among smear positive population in Uganda [30]. Predictive values reported in RIF mono resistance determination by both GeneXpert and LPA was high thus a better diagnostic tool for RIF resistance diagnosis.

This study reported a moderate agreement between GeneXpert MTB/RIF and culture (Kappa Value, 0.4109), and also LPA and culture (Kappa Value, 0.5914). Similarly, there was a moderate agreement between GeneXpert MTB/RIF and culture *(*Kappa Value*,* 0.5905) for Rifampicin mono resistance detection while, on the other hand there was a very good agreement between LPA and culture *(*Kappa Value*,* 0.8635) for detection of RIF mono-resistance. The current study reported a high agreement similar to what was reported in India, of 100% between MGIT960 and LPA results but only 64.4% agreement with GeneXpert MTB/RIF results [31], equally LPA reporting the same with the conventional DST at 96% in New Delhi [32]. Therefore, the study recommends use of either GeneXpert or LPA for TB detection and LPA for RIF mono-resistant.

Our results slightly vary from similar studies. A case example are the results that showed the sensitivity and specificity of the GeneXpert assay in *M. tuberculosis* samples from South Africa and Turkey to be 92.7–100% and 96.3–100%, respectively [21, 33–37]. In other areas such as Vietnam and Malaysia, similar values have been reported as 59 and 53%, respectively which is much lower than our findings [12, 14, 21] . The variations of the assay performance characteristics can be attributed to the geographical features of the sampling locations, differences in sampling method, MDR-TB and mutations on the *rpoB* gene in populations.

## Conclusion

In our context, when MGIT is used as the reference assay standard, Gene-Xpert has a better diagnostic performance characteristic than AFB Microscopy but lower when compared to the LPA. In addition, regarding RIF mono-resistance, LPA outperformed GeneXpert MTB/RIF and thus it is a better alternative to culture with regards to detection of RIF.

## Supplementary information

**Additional file 1.** Number of samples collected from the 34 counties.

## Data Availability

All data contained within the article and its Additional file 1.

## Abbreviations

AFB: Acid fast bacili
BHI: Brain heart infusion agar
INH: Isoniazid
LPA: Line probe assay
MDR-TB: Multi-drug resistance tuberculosis
MGIT: Mycobacterium growth indicator tube
MTB: *Mycobacterium tuberculosis*
NALC-NaOH: *N*-acetyl-l-cysteine-sodium citrate-NaOH
NTRL: National TB reference laboratory
RIF: Rifampicin
