# Supplementary material for: Comparison of GeneXpert and line probe assay for detection of Mycobacterium tuberculosis and rifampicin-mono resistance at the National Tuberculosis Reference Laboratory, Kenya
Source: BMC Infect Dis. 2019 Oct 15;19:852. doi: 10.1186/s12879-019-4470-9 (PMC6794895; doi:10.1186/s12879-019-4470-9)
Supplement: Supplementary file 1 — Additional file 1. Number of samples collected from the 34 counties. [file 12879_2019_4470_MOESM1_ESM.docx]

**Additional file 1: Number of samples collected from the 34 counties**

| **County** | **Frequency** | **Percent** |
| --- | --- | --- |
| **Baringo** | 1 | 0.3 |
| **Bomet** | 2 | 0.7 |
| **Bungoma** | 7 | 2.3 |
| **Embu** | 7 | 2.3 |
| **Garissa** | 3 | 1.0 |
| **Isiolo** | 1 | 0.3 |
| **Kajiado** | 6 | 2.0 |
| **Kakamega** | 3 | 1.0 |
| **Kiambu** | 20 | 6.5 |
| **Kilifi** | 9 | 2.9 |
| **Kirinyaga** | 17 | 5.6 |
| **Kitui** | 7 | 2.3 |
| **Laikipia** | 6 | 2.0 |
| **Machakos** | 18 | 5.9 |
| **Makueni** | 6 | 2.0 |
| **Marsabit** | 1 | 0.3 |
| **Meru** | 29 | 9.5 |
| **Mombasa** | 21 | 6.9 |
| **Murang'a** | 18 | 5.9 |
| **Nairobi** | 70 | 22.9 |
| **Nakuru** | 14 | 4.6 |
| **Nandi** | 1 | 0.3 |
| **Narok** | 8 | 2.6 |
| **Nyamira** | 1 | 0.3 |
| **Nyandarua** | 5 | 1.6 |
| **Nyeri** | 11 | 3.6 |
| **Samburu** | 1 | 0.3 |
| **Siaya** | 1 | 0.3 |
| **Taita Taveta** | 2 | 0.7 |
| **Tana River** | 1 | 0.3 |
| **Tharaka Nithi** | 2 | 0.7 |
| **Turkana** | 1 | 0.3 |
| **Vihiga** | 1 | 0.3 |
| **West Pokot** | 5 | 1.6 |

The table indicate the names of the counties and the amount of samples obtained from each to perform the validation
